# Exploring the population interaction of Przewalski’s gazelle (*Procapra przewalskii*) based on the variations in gut microbiota across diverse geographic populations

**DOI:** 10.3389/fmicb.2024.1439554

**Published:** 2024-08-21

**Authors:** Jingjie Zhang, Pengfei Song, Feng Jiang, Tongzuo Zhang

**Affiliations:** ^1^State Key Laboratory of Plateau Ecology and Agriculture, Qinghai University, Xining, China; ^2^Key Laboratory of Adaptation and Evolution of Plateau Biota, Northwest Institute of Plateau Biology, Chinese Academy of Sciences, Xining, China; ^3^University of Chinese Academy of Sciences, Beijing, China; ^4^Qinghai Provincial Key Laboratory of Animal Ecological Genomics, Xining, China

**Keywords:** Przewalski’s gazelle, interactions, gut microbiota, diversity, composition, function

## Abstract

The differences in gut microbiota among different populations, to a certain extent, reflect the degree of interaction between individuals within populations. To assess the interaction levels among several small populations of Przewalski’s gazelle (*Procapra przewalskii*) (*n* = 105, from seven different regions) based on differences in gut microbiota, we used the closely related Tibetan gazelle (*P. picticaudata*) (*n* = 52, from seven different regions) as a control. We then compared the gut microbial communities between different populations of the two species using high-throughput sequencing of the 16S rRNA gene. The results showed that within a 100 km geographical distance, the intergroup differences in relative abundance of dominant bacteria, α-diversity, β-diversity, and functional metabolism abundance were higher or significantly higher in Przewalski’s gazelle (narrowly distributed species) compared to the Tibetan gazelle (widely distributed species). Additionally, the proportion of shared OTUs between groups in Przewalski’s gazelle was significantly lower than in Tibetan gazelle (*p* < 0.05). Additionally, neutral community model results also showed lower dispersal limitation in the Tibetan gazelle compared to Przewalski’s gazelle. Therefore, based on the above results, we comprehensively speculate that the spatial interaction degree of Przewalski’s gazelle in different habitat patches is relatively low. This study, starting from the perspective of gut microbiota, adopts a non-genetic perspective or method to assess whether there is, or to what extent there is, close interaction between species populations.

## 1 Introduction

Amidst the ongoing global decline in biodiversity, urgent and critical attention is directed toward biodiversity conservation. The Qinghai-Tibet Plateau is a recognized global biodiversity hotspot, fostering numerous rare plant and animal species due to its unique geography and climate, making it a key area for global biodiversity research (Yang and Xia, 2008; Qiu et al., 2015). As human activities continue to expand and environmental changes intensify, the ecosystems of the Qinghai-Tibet Plateau face immense pressure (Shi et al., 2023). Strengthening the conservation of endangered species becomes an urgent necessity to protect the entire ecosystem and maintain biodiversity. In this process, particular emphasis on the protection of endangered species becomes crucial. Among them, Przewalski’s gazelle (*Procapra przewalskii*) is a typical globally endangered species in urgent need of protection. Facing numerous threats, it exhibits relatively low genetic diversity, and communication among its populations is relatively limited. Przewalski’s gazelle is not only one of the world’s most endangered species but also a unique species endemic to the Qinghai-Tibet Plateau (Shi et al., 2023). This species belongs to the genus *Procapra* in the subfamily Antilopinae of the family Bovidae, which is classified under the order Artiodactyla. It is a first-grade key protected wildlife species in China and is listed as Critically Endangered (CR) in the China Vertebrate Red List (Jiang et al., 2016) and assessed as Endangered (EN) by the International Union for Conservation of Nature (IUCN) Red List. Historically, Przewalski’s gazelle was widely distributed in the western part of China. However, rapid urbanization, extensive use of infrastructure such as roads and railways, and overexploitation of natural resources have led to a drastic decline in the population of this species. These human activities have fragmented the suitable habitat for the species into small and relatively isolated patches (Jiang et al., 2000; Li et al., 2012). Nevertheless, it remains unclear whether there is communication between populations of Przewalski’s gazelle in different habitat patches.

Currently, with the rapid development of high-throughput sequencing technologies, the study of gut microbiota is widely applied in conservation biology research, particularly for endangered species. Gut microbiota coevolves with the host, forming a complex microbial ecosystem crucial for the host’s health, which plays a vital role in essential physiological activities such as food digestion, nutrient absorption, and immune regulation (Dethlefsen et al., 2007; Sampson and Mazmanian, 2015). Moreover, gut microbiota is influenced by external factors such as genetic background, age, gender, food composition, geographic environment, and seasonal variations. Existing studies have indicated that, despite the host genotype being considered a crucial factor in shaping the gut microbiota, the external environment can alter the composition and diversity of the host’s gut microbiota (Spor et al., 2011; Wasimuddin et al., 2017; Zhao et al., 2018; Guo et al., 2019). The dietary composition of most herbivores is adapted to the plant diversity and composition in their environment, and differences in food resources are the most direct factors influencing the gut microbiota of the host (Muegge et al., 2011; Scott et al., 2013; Flint et al., 2015; Graf et al., 2015; Sandhu et al., 2017). In the Tibetan, Yunnan, and Guizhou regions of China, six different wintering sites for the black-necked crane (*Grus nigricollis*) showed significant differences in gut microbiota alpha diversity, beta diversity, and partial dominant bacteria, with various food resources potentially playing a crucial role in these variations (Wang et al., 2020). Additionally, diverse food resources and climatic environments have resulted in significant differences in alpha (α) diversity, beta (β) diversity, core bacterial relative abundance, and functionality of the gut microbiota in six different regions of rhesus macaques (*Macaca mulatta*) (Zhao et al., 2018).

Based on our preliminary investigations, Przewalski’s gazelle is currently found only in the vicinity of Qinghai Lake (Zhang et al., 2021a,b). Apart from Shengge Township, other regions are relatively close geographically, with variations in habitat types (Zhang et al., 2022). The differences in vegetation types directly influence the availability of different food resources. Assuming relatively frequent species dispersal and foraging exchanges between different habitat patches of Przewalski’s gazelle, the intergroup differences in the composition, diversity, and functionality of their gut microbiota might be relatively small. Additionally, the closely related Tibetan gazelle (*P. picticaudata*), which is widely distributed across the Qinghai-Tibet Plateau, serves as a suitable control species. This study aims to explore the spatial interactions among small populations of Przewalski’s gazelle from different regions in terms of gut microbiota composition, diversity, and functionality. Fresh fecal samples were collected simultaneously from seven small geographical populations of Przewalski’s gazelle, covering almost all current distribution areas of the species. Using the widely distributed Tibetan gazelle as a control, this study investigates the degree of interaction among small populations of Przewalski’s gazelle based on gut microbiota composition, dominant bacteria, α-diversity, β-diversity, and metabolic functions across different regions.

## 2 Materials and methods

### 2.1 Collection and processing of Przewalski’s gazelle fecal samples

During the winter period from late 2018 to early 2019, a total of 105 fecal samples were collected from Przewalski’s gazelle in seven different regions, namely WY (Wayu township), ND (Niaodao scenic area), GH (Ganzihe and Haergai townships), NC (Nongchang), SD (Shadao scenic area), KT (Ketu township), and JX (Jiangxigou Rescue Station), based on their ecological characteristics and current spatial distribution. These regions correspond to those mentioned in our previously published paper and essentially cover the current existing distribution areas of Przewalski’s gazelle. The abbreviations of the distribution locations here were consistent with those used in our previous study (Zhang et al., 2022).

For comparative analysis, in the winter of 2019, we collected a total of 52 fresh fecal samples from Tibetan gazelles in seven different regions in Qinghai Province (locations named PWD, PWF, PWG, PWH, PWI, PWN, and PWO). During the sampling process, each sample was collected using disposable PE gloves to prevent cross-contamination. The experimental samples were placed in sterile, self-sealing bags, and relevant information (including sample number and sampling date) was recorded. Latitude and longitude information were recorded using a handheld GPS recorder (GPSMAP 63csx, Garmin, China). Fecal samples were placed in dry ice, a portable refrigerator, or wrapped in aluminum foil and rapidly transferred to a liquid nitrogen tank for preservation. Upon return to the laboratory, all samples were stored in a −80°C ultra-low temperature freezer for subsequent DNA extraction from the feces.

### 2.2 DNA extraction and quality assessment from fecal samples of Przewalski’s gazelle and Tibetan gazelle

Following the instructions provided by the DNA extraction kit (E.Z.N.A.^®^ Soil DNA Kit, Omega Bio-Tek, United States), we separately extracted total DNA from fecal samples of Przewalski’s gazelle and Tibetan gazelle to obtain their respective total DNA samples. The purity and concentration of the total DNA were assessed using a microvolume spectrophotometer (NanoDrop2000, Thermo Fisher Scientific, United States), and OD260/280 and OD260/230 values were recorded. Additionally, the integrity of the total DNA was evaluated through 1% agarose gel electrophoresis (Biowest agarose, Biowes, Spain) at 5 V/cm for 20 min.

### 2.3 Amplification and purification of 16S rRNA gene

This study selected universal primers to amplify the V4-V5 variable region of the bacterial 16S rRNA gene from Przewalski’s gazelle and Tibetan gazelle fecal samples. The primer information was as follows: 515F: 5′-GTGCCAGCMGCCGCGG-3′; 907R: 5′-CCGT CAATTCMTTTRAGTTT-3′. The PCR reaction system (20 μL) was composed of 4 μL 5× TransStart FastPfu Buffer, 2 μL 2.5 mM dNTPs, 0.8 μL of each upstream and downstream primers (5 μM), 0.4 μL TransStart FastPfu DNA Polymerase, and 10 ng template DNA (using ddH_2_O as a blank control), adjusted to a total volume of 20 μL.

The PCR amplification program was as follows: 95°C for 3 min; 27 cycles of 95°C for 30s, 55°C for 30s, 72°C for 45 s; 72°C for 10 min; and storage at 4°C. The PCR products from three replicates were mixed, and 3 μL of the PCR product was subjected to 2% agarose gel electrophoresis. The PCR products were purified using the DNA Gel Extraction Kit (Axygen Biosciences, Axygen, United States), and the purified PCR products were quantified using a microplate fluorometer (Quantus^™^ Fluorometer).

### 2.4 Sequencing data processing and bioinformatics analysis

#### 2.4.1 Sequence data processing

The raw reads of the samples were subjected to quality control using Trimmomatic (version 0.39) (Bolger et al., 2014) to obtain high-quality sequences (clean reads). FLASH (Fast Length Adjustment of SHort reads) software was employed to perform paired-end assembly based on the overlapping relationship between forward and reverse sequences (Magoč and Salzberg, 2011), resulting in raw tags sequences. The UCHIME algorithm, with reference to the database, was applied to identify and remove chimeric sequences, yielding clean tags sequences. The Uparse software (version 7.1, http://drive5.com/uparse/) was used to eliminate unique sequences without duplicates from clean tags (Costello et al., 2009; Edgar, 2013), and clustering at 97% sequence similarity was performed to obtain Operational Taxonomic Units (OTUs) representative sequences.

The SILVA 138/16S bacterial annotation database was constructed using the RESCRIPt (reference sequence annotation and curation pipeline) software (Robeson et al., 2021) and the SILVA rRNA database (version 138) (Quast et al., 2012; Glöckner et al., 2017). The OTU representative sequences were taxonomically annotated using the RDP Classifier software with a confidence threshold set at 0.8. Multiple sequence alignment of OTU representative sequences was performed using the MAFFT software (Katoh and Standley, 2013). The phylogenetic tree was constructed with IQ-TREE (Kalyaanamoorthy et al., 2017; Minh et al., 2020), and the substitution model was automatically assessed and selected by ModelFinder (Kalyaanamoorthy et al., 2017).

#### 2.4.2 Statistical analysis and data visualization

Annotating OTUs for taxonomic classification, we recorded the abundance information for each annotation. Dilution curves were plotted after rarefaction based on the minimum sample sequence number. Relative abundance bar charts (at the phylum and genus levels) were generated. Venn diagrams were employed to analyze the core and unique genera and phyla of gut microbiota in Przewalski’s gazelle across different regions as well as in different regions of the Tibetan gazelle. Cluster heatmaps were constructed to analyze the compositional differences in gut microbiota among Przewalski’s gazelle populations across different regions as well as in different regions of Tibetan gazelle.

In the analysis of α-diversity, the Sobs index (observed OTUs, richness index), Shannon index, Chao1 index (richness index), and PD (phylogenetic diversity, diversity index) were selected to assess the diversity of gut microbiota in Przewalski’s gazelle across different regions as well as in different regions of the Tibetan gazelle. The α-diversity indices were calculated using Qiime software (version 1.9.1). In β-diversity analysis, principal coordinates analysis (PCoA) and non-metric multidimensional scaling (NMDS) were employed and Bray-Curtis, unweighted unifrac, and weighted unifrac similarity distance algorithms were used to assess the β-diversity of gut microbiota in Przewalski’s gazelle across different regions as well as in different regions of Tibetan gazelle. ANOSIM (Analysis of similarities) and Adonis analysis (Permutational MANOVA) were conducted to perform differential testing analysis of gut microbiota composition across different regions (Oksanen et al., 2019).

The differences in the abundance of dominant phyla and genera in the gut microbiota of Przewalski’s gazelle across different regions as well as in different regions of Tibetan gazelle were analyzed using the Wilcoxon rank-sum test. To identify differentially abundant species among groups, LEfSe was utilized, and Linear Discriminant Analysis (LDA score) was employed to quantify the impact of different species on the distinction between groups. Functional predictions of 16S rRNA data were performed using PICRUSt (Phylogenetic Investigation of Communities by Reconstruction of Unobserved States). Standardized OTU abundance tables were aligned against the KEGG database and EggNOG database to obtain functional abundance information at multiple levels, including KEGG Orthology (KO), pathways, and COG functions. Wilcoxon rank-sum tests was then conducted to analyze intergroup differences in metabolic functions across different regions of Przewalski’s gazelle (as well as in different regions of the Tibetan gazelle). Taking Tibetan gazelle as a control group, Przewalski’s gazelle and Tibetan gazelle from different regions were selected within the same distance range. From the perspectives of gut microbiota composition, shared OTU proportions, dominant phyla, dominant genera, α-diversity analysis, β-diversity analysis, and metabolic functions related to different substances, the study aimed to explore the differences in gut microbiota composition and functionality across different regions of Przewalski’s gazelle.

## 3 Results

### 3.1 The common OTU proportion between two species

The analysis indicated that the proportion of shared OTUs in the gut microbiota of Przewalski’s gazelle across different regions ranges between 70 and 80%, while in the gut microbiota of Tibetan gazelle across different regions, the shared OTU proportion ranges between 85 and 90% (Table 1). Within a 100 km range, the Wilcoxon rank-sum test analysis revealed that the shared OTU proportion of gut microbiota in Przewalski’s gazelle across different regions was significantly lower than that in Tibetan gazelle across different regions (*p* < 0.001).

**Table 1:** Comparative analysis of the number of common/endemic OTUs of Przewalski’s gazelle and Tibetan gazelle in different areas.

Groups	Straight line distance (km)	Actual distance (km)	Shared OTUs	Specific OUT of Group 1	Specific OUT of Group 2	Proportion of shared OTUs (%)	Proportion of non-shared OTUs (%)
KT-SD	12.58	15.24	2,327	335	431	75.23	24.77
NC-GH	14.09	17.14	2,727	343	307	80.75	19.25
GH-SD	42.41	45.08	2,470	564	288	74.35	25.65
NC-SD	44.73	51.56	2,471	599	287	73.61	26.39
JX-KT	38.85	57.14	2,319	636	343	70.32	29.68
KT-GH	53.64	58.41	2,439	223	595	74.88	25.12
JX-SD	39.03	64.76	2,362	593	396	70.49	29.51
KT-NC	57.09	66.03	2,433	229	637	73.75	26.25
ND-WY	75.76	92.70	2,313	284	646	71.32	28.68
NC-ND	43.05	96.51	2,345	725	252	70.59	29.41
PWH-PWO	70.02	70.02	2,208	115	171	88.53	11.47
PWD-PWO	71.31	71.31	2086	91	293	84.45	15.55
PWF-PWO	78.05	78.05	2,132	113	247	85.55	14.45
PWF-PWN	78.27	78.27	2055	190	201	84.01	15.99
PWG-PWO	79.89	79.89	2,187	111	192	87.83	12.17
PWH-PWN	87.39	87.39	2,117	206	139	85.99	14.01
PWI-PWN	92.71	92.71	2045	175	211	84.12	15.88

### 3.2 Analysis of differences between α diversity groups of gut microbiota in two species

In this study, the Shannon index, Sobs index, ACE index and Chao1 index were selected to conduct research on the α diversity of gut microbiota in Przewalski’s gazelle and Tibetan gazelle in different regions. Among them, the Shannon index reflects the diversity of gut microbiota, while the latter three reflect the richness. The results of the intergroup difference analysis of the alpha diversity of the gut microbiota in Przewalski’s gazelle between any two regions within a geographical distance of 100 km showed that, except for the KT and SD groups (*d* = 15.24 km), NC and GH groups (*d* = 17.14 km), there were significant differences in the alpha diversity of gut microbiota in Przewalski’s gazelle among the other seven groups (Table 2) (Zhang et al., 2022). Using the aforementioned comparative analysis of the Tibetan gazelles in different areas within the 100 km range, the study pointed out that there was no significant difference in the four α-diversity indicators between different groups on the whole (Table 2). Overall, it can be seen that within the same distance range, the intergroup difference in the alpha diversity of the gut microbiota in Przewalski’s gazelle in different regions was higher than that of Tibetan gazelle in different regions.

**Table 2:** Comparison of *p* values of α diversity in different regions of Przewalski’s gazelle and Tibetan gazelle within 100 km range.

No.	Groups	Shannon	Sobs	ACE	Chao1
1	KT-SD	0.901 ns	0.455 ns	0.300 ns	0.619 ns
2	NC-GH	0.300 ns	0.534 ns	0.590 ns	0.868 ns
3	GH-SD	<0.001***	<0.001***	<0.001***	<0.001***
4	NC-SD	<0.001***	<0.001***	<0.001***	<0.001***
5	JX-KT	<0.001***	<0.001***	<0.001***	<0.001***
6	KT-GH	<0.001***	<0.001***	<0.001***	<0.001***
7	JX-SD	<0.001***	<0.001***	<0.001***	<0.001***
8	KT-NC	<0.001***	<0.001***	<0.001***	<0.001***
9	ND-WY	0.023 *	<0.001***	<0.001***	<0.001***
10	NC-ND	<0.001***	<0.001***	<0.001***	<0.001***
11	PWH-PWO	0.427 ns	0.930 ns	0.930 ns	0.930 ns
12	PWD-PWO	0.768 ns	0.008**	0.999 ns	0.953 ns
13	PWF-PWO	0.999 ns	0.138 ns	0.916 ns	0.916 ns
14	PWF-PWN	0.520 ns	0.520 ns	0.721 ns	0.284 ns
15	PWG-PWO	0.751 ns	0.244 ns	0.525 ns	0.999 ns
16	PWH-PWN	0.377 ns	0.051 ns	0.263 ns	0.317 ns
17	PWI-PWN	0.561 ns	0.651 ns	0.999 ns	0.747 ns

### 3.3 Analysis of differences between β diversity groups of gut microbiota of two species

**Figure 1:**
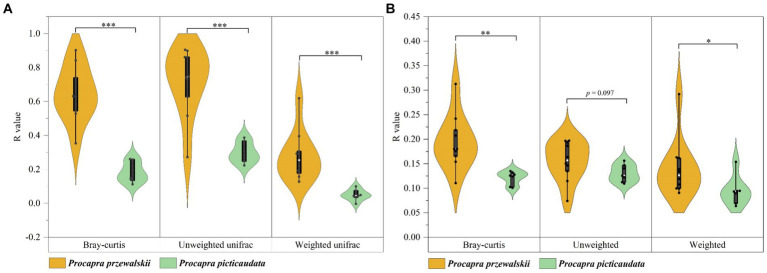
Analysis of differences in gut microbiota composition in different regions of Przewalski’s gazelle and the Tibetan gazelle within 100 km based on ANOSIM **(A)** and Adonis **(B)** tests. *p < 0.05; **p < 0.01; ***p < 0.001.

Taking Tibetan gazelles whose geographical distance between populations is within 100 km as a comparison, the ANOSIM and Adonis tests based on Bray-Curtis and unweighted uniFrac distance algorithms pointed out that although there were significant differences in the gut microbiota composition of Tibetan gazelles in different regions, mainly reflected as small or extremely small differences. The ANOSIM and Adonis tests based on the weighted uniFrac distance algorithm pointed out that there were no significant differences in the composition of gut microbiota of Tibetan gazelles in different regions. However, there were significant differences among the populations of Przewalski’s gazelle based on the above three algorithms and two tests [Supplementary Table S1, the values pertaining to the intergroup difference analysis of Przewalski’s gazelle in the table were sourced from our preliminary study (Zhang et al., 2022)]. In addition, this study showed that through the Wilcoxon rank sum test of the R value (ANOSIM test) and *R*^2^ value (Adonis test), the difference in β-diversity of the gut microbiota in Przewalski’s gazelle among different regions was significantly higher than that in Tibetan gazelle (Figure 1).

### 3.4 Analysis of differences between the dominant bacteria of gut microbiota in the two species

**Figure 2:**
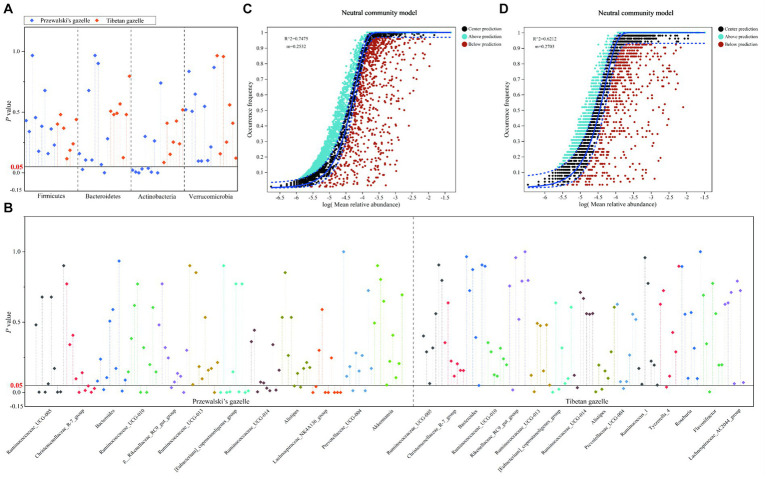
Difference analysis of the dominant phyla **(A)** and genus **(B)** of gut microbiome in different regions of Przewalski’s gazelle and Tibetan gazelle. NCM analysis of Przewalski’s gazelle **(C)** and Tibetan gazelle **(D)**.

The Wilcoxon rank-sum test was used to analyze the differences between the dominant bacterial groups between each pair of groups of Przewalski’s gazelle and Tibetan gazelle within a 100 km range. At the phylum level, there was no significant difference in the relative abundance of the phyla Firmicutes and Verrucomicrobia of gut microbiota in Przewalski’s gazelle in different regions. The relative abundance of phylum Bacteroidetes in NC and GH groups, ND and WY groups showed significant differences. There were significant differences in the relative abundance of phylum Actinobacteria between KT and SD groups, NC and GH groups, GH and SD groups, NC and SD groups, KT and GH groups, JX and SD groups, and ND and WY groups (Figure 2A). Meanwhile, there was no significant difference in the relative abundance of the four dominant bacterial phyla of gut microbiota in Tibetan gazelles in different regions (Figure 2A).

Among the 12 dominant bacterial genera in Przewalski’s gazelle, about 30.8% (37/120) showed significant differences in the relative abundance of dominant bacterial genera between groups. Among the 15 dominant bacterial genera in Tibetan gazelle, about 9.5% (10/105) showed significant differences in the relative abundance of dominant bacterial genera between groups. Overall, it can be seen that the differences between the dominant bacterial groups of the gut microbiota in Przewalski’s gazelle in different areas within a 100 km range was higher than those in Tibetan gazelle in different areas (Figure 2B).

**Figure 3:**
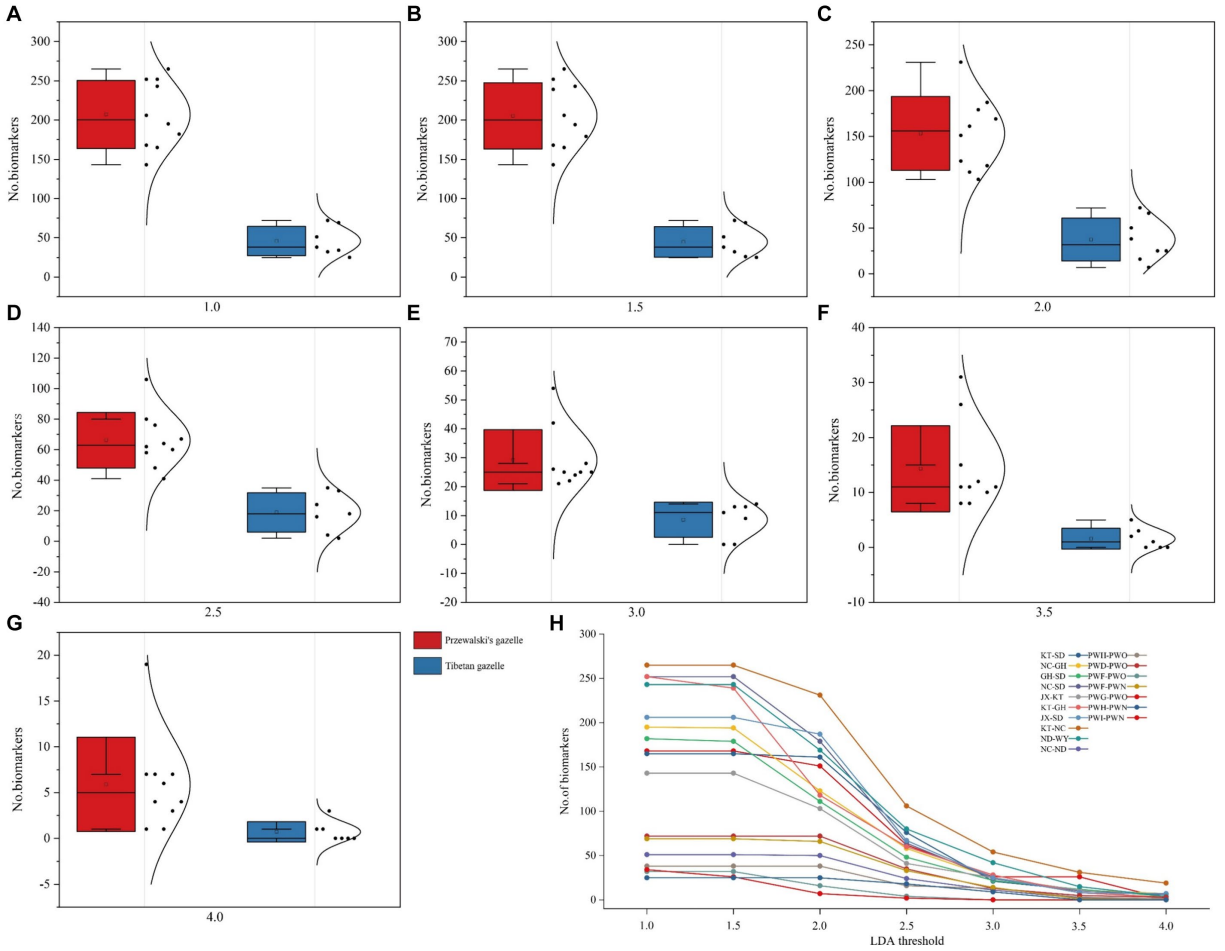
Lefse analysis of the difference in the number of biomarker bacteria in the gut microbiome of Przewalski’s gazelle and Tibetan gazelle in different regions. **(A–G)** Represent the difference in the number of biomarker bacteria when lad ranges from 1.0 to 4.0, respectively. **(H)** Represents the number of biomarkers of each lad value.

The biomarker bacteria in Przewalski’s gazelle and Tibetan gazelle in different areas were identified through LEfSe analysis (*p* < 0.05). Seven different thresholds were set for the LDA score, and the Wilcoxon rank-sum test was used to conduct intergroup difference analysis of the number of biomarker bacteria between each pair of groups. The results showed that the number of biomarker bacteria with significant differences between groups in Przewalski’s gazelle in different regions was significantly higher than that in Tibetan gazelles in different regions (Figure 3; Table 2).

### 3.5 Analysis of neutral community model of Przewalski’s gazelle and Tibetan gazelle

The analysis results of the neutral community model (NCM) showed that the *R*^2^ of Przewalski’s gazelle and Tibetan gazelle were both higher than 0.5, proving that the construction of gut microbiota communities of the two species was greatly affected by stochastic processes. However, the m value of Tibetan gazelle was higher than that of Przewalski’s gazelle, proving that Tibetan gazelle was less restricted in dispersal than Przewalski’s gazelle (Figures 2C,D).

### 3.6 Analysis of metabolic function differences between the dominant gut microbiota groups of the two species

**Figure 4:**
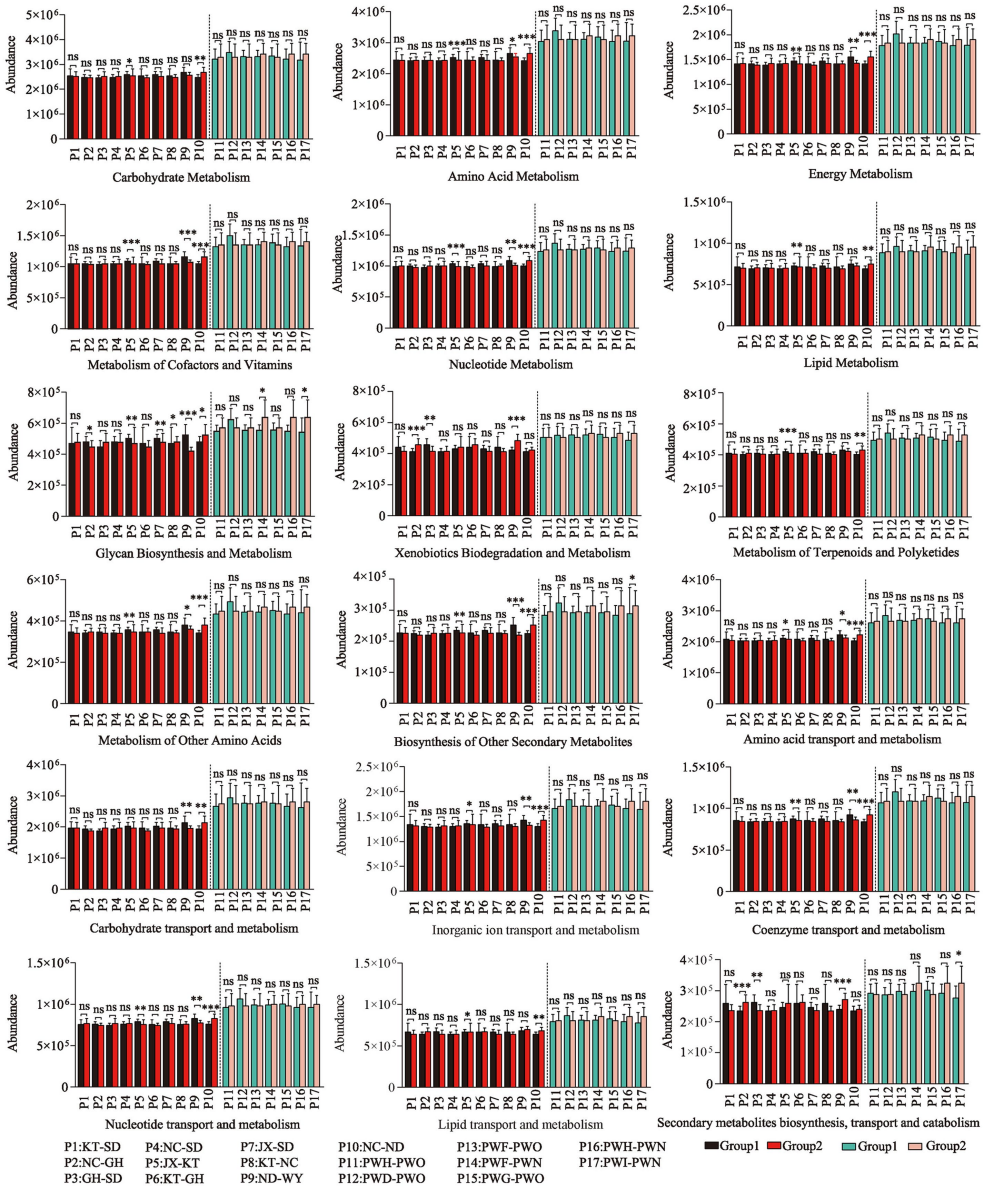
Analysis of differences in metabolic function of gut microbiome in different regions in Przewalski’s gazelle and the Tibetan gazelle based on KEGG database and EggNOG database. *p < 0.05; **p < 0.01; ***p < 0.001.

The Wilcoxon rank-sum test was used to conduct intergroup difference analysis of metabolic functions between each pair of groups of Przewalski’s gazelle and Tibetan gazelle within a 100 km range. In Przewalski’s gazelle, 28.9% (52/180) of substances showed significant differences in metabolic function abundance values between groups, while in Tibetan gazelle, about 3.2% (4/126) of substances showed significant differences in metabolic function abundance values among groups. Overall, it can be seen that the abundance of material metabolic functions of the gut microbiota in Przewalski’s gazelle in different areas within a 100 km range was higher than that in Tibetan gazelles in different areas (Figure 4).

## 4 Discussion

Przewalski’s gazelle has a narrow distribution range around the world, and the geographical distances between several distribution areas around Qinghai Lake are relatively short. However, our previous study has indicated that the gut microbiota of Przewalski’s gazelle distributed in such geographicallyshort regions have different levels of α-diversity, while there were significant differences in β-diversity and predictive function (Zhang et al., 2022). What is the reason for this difference? First of all, dietary differences may be the main reason, as there is evidence that although several distribution areas of Przewalski’s gazelle are close to each other, their vegetation types are quite different (Zhang et al., 2022). This means that if Przewalski’s gazelles do not migrate or communicate among several populations, the food resources they can choose are relatively limited and highly different. In addition, there may be a lack of microbial transmission pathways between individuals who do not communicate or have low levels of communication, thus possibly leading to low homogeneity among gut community members in several distribution groups (Moeller et al., 2016). Other studies have shown that through mutual transfer experiments of salamander larvae between different habitats, habitat-switching salamanders either undergo a shift in gut microbiota that converges with the native species or that the predicted functions of their gut microbiota converge with those of larvae from the destination habitat (Bletz et al., 2016). It has also been shown that microbial taxon similarity is higher in hosts living in groups with high population densities and frequent contact between individuals (Amato, 2013). For example, cohabiting, genetically unrelated partners have more similar gut bacterial communities than individuals living in different households (Yatsunenko et al., 2012; Song et al., 2013; Mosites et al., 2017). Combining the above research results, this study shows that the degree of communication among several populations of Przewalski’s gazelle was relatively low. To further strengthen our evidence, we used the Tibetan gazelle, a closely related specie of Przewalski’s gazelle andspecies with the ability to migrate long distances on the Qinghai-Tibet Plateau, as a control group. Based on the relatively similar geographical distance between the two species, we compared the gut microbiota between them. The size of the difference was used to judge the degree of communication between Przewalski’s gazelle populations.

Considering that the distance between the populations is too long, it may result in low inter-population communication and large differences in food composition. We chose the distance between the respective populations of the two species to be within 100 km. Therefore, in the long distance, it is not appropriate to carry out this research among populations. Previous research has indicated that there were relatively frequent exchanges between different populations in Maduo County, and that this species had the habit of migrating long distances for food (Lu, 2005). This may be the main factor leading to the lack of significant differences in the composition of gut microbiota among different populations in Maduo County. However, this study showed that significant differences in gut microbiota between Przewalski’s gazelle populations were evident in both diversity and function. As mentioned above, this difference is likely caused by differences in food composition, and most studies have also shown that food is the most important external factor causing differences in host gut microbiota (Maukonen and Saarela, 2015; Miyake et al., 2015; Perry et al., 2022). According to our survey, in addition to the relatively diverse and large differences in vegetation types in different distribution areas, Przewalski’s gazelles also receive a certain proportion of artificial supplementary feeding in the Ganzihe-Hargai area (GH group) in winter. In the captive environment, Przewalski’s gazelles in this area may obtain food resources in addition to the existing vegetation, and some of these resources are provided through artificial supplementary feeding. This is also one of the ways that Przewalski’s gazelles in different distribution areas can obtain different food resources.

In summary, we compared the differences in gut microbiota between different populations of the two species and concluded that the degree of communication between Przewalski’s gazelle populations is relatively low. This can also be concluded from the large differences in vegetation types between different populations of Przewalski’s gazelle. This difference is likely caused by feeding on different vegetation. In addition, other studies related to molecular genetics also indicated that the degree of communication between Przewalski’s gazelle populations is low (Lei et al., 2003; Yang and Jiang, 2011; Yu et al., 2017). This study starts from the perspective of intestinal microorganisms and uses a non-genetic perspective or method to address whether, or to what extent, there is close communication between species populations. In follow-up research, we will further verify the results by studying the food composition of a small population of Przewalski’s gazelles and by using collars. In the future, further screening will be conducted to improve the diversity of the gut microbiota in Przewalski’s gazelle. This study provides a theoretical basis for the protection of this endangered species.

## Data Availability

The datasets generated for this study can be found in the raw sequences of 16S rRNA gene that were available in the NCBI Sequence Read Archive under BioProject accession numbers PRJNA722780 and PRJNA1140140.
