# Supplementary material for: Exploring the population interaction of Przewalski’s gazelle (Procapra przewalskii) based on the variations in gut microbiota across diverse geographic populations
Source: Front Microbiol. 2024 Aug 21;15:1439554. doi: 10.3389/fmicb.2024.1439554 (PMC11371741; doi:10.3389/fmicb.2024.1439554)
Supplement: Supplementary file 1 [file Table_1.DOCX]

Supplementary Material

# Supplementary Tables

| Distance algorithm | Bray-curtis | | | | Unweighted unifrac | | | | Weighted unifrac | | | |
| --- | --- | --- | --- | --- | --- | --- | --- | --- | --- | --- | --- | --- |
| Statistical test | ANOSIM | | Adonis | | ANOSIM | | Adonis | | ANOSIM | | Adonis | |
| Value | R | *P* | R^2^ | *P* | R | *P* | R^2^ | *P* | R | *P* | R^2^ | *P* |
| KT-SD | 0.5455 | 0.001 | 0.1652 | 0.001 | 0.5162 | 0.001 | 0.1147 | 0.001 | 0.1274 | 0.015 | 0.0905 | 0.009 |
| NC-GH | 0.3532 | 0.001 | 0.1105 | 0.001 | 0.2723 | 0.001 | 0.0741 | 0.002 | 0.1574 | 0.002 | 0.0996 | 0.007 |
| GH-SD | 0.6316 | 0.001 | 0.1801 | 0.001 | 0.7433 | 0.001 | 0.1465 | 0.001 | 0.29 | 0.001 | 0.1389 | 0.001 |
| NC-SD | 0.63 | 0.001 | 0.1803 | 0.001 | 0.7471 | 0.001 | 0.1505 | 0.001 | 0.1926 | 0.001 | 0.1078 | 0.002 |
| JX-KT | 0.8423 | 0.001 | 0.2419 | 0.001 | 0.8611 | 0.001 | 0.197 | 0.001 | 0.3956 | 0.001 | 0.1616 | 0.001 |
| KT-GH | 0.5292 | 0.001 | 0.154 | 0.001 | 0.6281 | 0.001 | 0.1346 | 0.001 | 0.1787 | 0.003 | 0.0991 | 0.007 |
| JX-SD | 0.7392 | 0.001 | 0.2075 | 0.001 | 0.9048 | 0.001 | 0.1966 | 0.001 | 0.306 | 0.001 | 0.1337 | 0.001 |
| KT-NC | 0.5807 | 0.001 | 0.1759 | 0.001 | 0.7305 | 0.001 | 0.1633 | 0.001 | 0.2188 | 0.001 | 0.1192 | 0.003 |
| ND-WY | 0.9027 | 0.001 | 0.3127 | 0.001 | 0.8987 | 0.001 | 0.1933 | 0.001 | 0.6192 | 0.001 | 0.292 | 0.001 |
| NC-ND | 0.72 | 0.001 | 0.2188 | 0.001 | 0.8612 | 0.001 | 0.1865 | 0.001 | 0.2927 | 0.001 | 0.163 | 0.001 |
| PWH-PWO | 0.1451 | 0.032 | 0.1008 | 0.013 | 0.2233 | 0.011 | 0.1124 | 0.006 | 0.0484 | 0.173 | 0.0688 | 0.278 |
| PWD-PWO | 0.1678 | 0.087 | 0.1276 | 0.011 | 0.268 | 0.012 | 0.1256 | 0.007 | 0.0363 | 0.29 | 0.0947 | 0.214 |
| PWF-PWO | 0.1924 | 0.031 | 0.1318 | 0.005 | 0.3868 | 0.006 | 0.156 | 0.006 | 0.0432 | 0.26 | 0.0932 | 0.18 |
| PWF-PWN | 0.1124 | 0.133 | 0.1242 | 0.06 | 0.2487 | 0.043 | 0.1467 | 0.014 | 0.0437 | 0.267 | 0.0898 | 0.36 |
| PWG-PWO | 0.1362 | 0.066 | 0.1258 | 0.01 | 0.3662 | 0.007 | 0.1404 | 0.005 | 0.0738 | 0.171 | 0.1538 | 0.061 |
| PWH-PWN | 0.2607 | 0.015 | 0.1025 | 0.04 | 0.3057 | 0.012 | 0.1095 | 0.008 | 0.0995 | 0.154 | 0.0636 | 0.441 |
| PWI-PWN | 0.2578 | 0.031 | 0.1348 | 0.014 | 0.2839 | 0.017 | 0.1223 | 0.014 | -0.0029 | 0.471 | 0.0942 | 0.278 |

**Supplementary Table 1** PCoA analysis at the OTU level between each two groups based on three distance algorithms with Anosim and Adonnis

**Supplementary Table 2** The number of biomarker bacteria in the gut microbiome of Przewalski’s gazelle and Tibetan gazelle in different regions

| LAD | 1.0 | 1.5 | 2.0 | 2.5 | 3.0 | 3.5 | 4.0 |
| --- | --- | --- | --- | --- | --- | --- | --- |
| KT-SD | 168 | 168 | 151 | 62 | 26 | 26 | 1 |
| NC-GH | 165 | 165 | 161 | 76 | 21 | 11 | 7 |
| GH-SD | 195 | 194 | 123 | 58 | 25 | 11 | 7 |
| NC-SD | 182 | 179 | 111 | 48 | 22 | 12 | 4 |
| JX-KT | 252 | 252 | 179 | 64 | 24 | 10 | 6 |
| KT-GH | 143 | 143 | 103 | 41 | 25 | 11 | 1 |
| JX-SD | 252 | 239 | 118 | 60 | 28 | 8 | 3 |
| KT-NC | 206 | 206 | 187 | 67 | 25 | 8 | 7 |
| ND-WY | 265 | 265 | 231 | 106 | 54 | 31 | 19 |
| NC-ND | 243 | 243 | 169 | 80 | 42 | 15 | 4 |
| PWH-PWO | 51 | 51 | 50 | 24 | 11 | 2 | 1 |
| PWD-PWO | 38 | 38 | 38 | 16 | 13 | 3 | 1 |
| PWF-PWO | 72 | 72 | 72 | 35 | 13 | 5 | 3 |
| PWF-PWN | 32 | 32 | 16 | 4 | 0 | 0 | 0 |
| PWG-PWO | 69 | 69 | 66 | 33 | 14 | 1 | 0 |
| PWH-PWN | 34 | 26 | 7 | 2 | 0 | 0 | 0 |
| PWI-PWN | 25 | 25 | 25 | 18 | 9 | 0 | 0 |
